# The Anatomy and Dislocation of the Shoulder-joint

**Published:** 1879-05

**Authors:** Wm. F. Sheehan

**Affiliations:** Rochester, N. Y.


### BUFFALO
### Medical and Surgical Journal.

VOL. XVIII. MAY, 1879. No. 10.

### Original Communications.

—:o:—

*ART. I.—The Anatomy and Dislocation of the Shoulder-Joint.* A paper read before the Rochester Pathological Society. By WM. F. SHEEHAN, M. D., Rochester, N. Y.

The shoulder-joint belongs to the class enarthrodial, i. e., it is composed of a ball and socket—the ball being the hemispherical head of the humerus, and the socket—the shallow glenoid fossa of the scapula. Tipping these bony structures, and serving as a protective cushion, is a layer of cartilage wafer-like in thickness, and holding them together, at the same time that it allows a certain laxity and range of motion, is a saccular fibrous structure, the capsular ligament. Finally there is a synovial membrane which lines the capsular ligament and is reflected therefrom on the articular cartilages. Thus, bone, cartilage, synovial membrane and capsule are the essentials—the *sine qau non* so to speak, of every joint. But there is need of tendons and ligaments, so called accessories, to complete the structure of a joint, and forthwith nature, always complete in her handiwork, supplies them. In this category the shoulder-joint presents for examination the glenoid ligament, the coraco-humeral ligament, and the tendons of insertions of the supraspinatus, infraspinatus, teres minor, and subscapularis muscles. As I regard these structures of vast importance in connection with shoulder dislocations, I shall claim for each as I describe it your individual attention.

I follow Gray:—The glenoid ligament is a firm fibrous band attached round the margin of the glenoid cavity; it deepens the cavity for articulation and protects the edge of the bone. It hugs the head of the humerus holding it in gentle grasp, and thereby helps to prevent luxation, or more probably sub-luxation, during the freest motions of the joint. The coraco-humeral or accessory ligament is a broad band which strengthens the upper and inner aspect of the capsule. It arises from the outer border of the coracoid process and extends obliquely downwards and outwards to the front of the great tuberosity of the humerus, being blended with the tendon of the supraspinatus muscle. This ligament is entirely united to the capsular in the greater part of its extent. The coraco-humeral may be termed Y ligament of the shoulder-joint. Between it and ilio-femoral ligament there is a striking analogy in mode of origin, distribution and insertion; and the time may come when the coraco-humeral ligament will play as important a role in relation to shoulder dislocations as the ilio-femoral does in the hip dislocations. Future investigation must of course settle this question. I myself have done some work along this line, but owing to a mishap in dissecting, the result was not determinate enough to warrant publication.

The tendon of the supraspinatus blends with and strengthens the capsule above and in front as it runs from the supraspinous fossa to be inserted into the major tuberosity; and the tendons of the infraspinatus and teres minor serve in the same capacity as they run from the infraspinous fossa and axillary border of the scapula over the back part of the capsule to be inserted into the major tuberosity near the attachment of the supraspinatus; whilst the tendon of the subscapularis strengthens the capsule in front in the neighborhood of the major tuberosity. He who has ever made a careful dissection of the shoulder-joint knows full well what nice touches of the scalpel are required to separate these tendons from the capsule. They seem to form, taken together, a capsule around the capsule, super-imposed like plates of steel on the part of a man-of-war where there is most need of the greatest strength. From this anatomical distribution I have deduced the conclusion: That an ordinary or rather an ordinary extraordinary force is not sufficient to rupture the capsule above, behind, or before—the points of contact with the capsule of the tendons of the supra-spinatus, infraspinatus, teres minor, and subscapularis muscles;—and there being impossibility of capsular rupture, there must needs be impossibility of dislocation in these directions. There is no need of controversy on this point, for I doubt if post mortem examination of a dislocated shoulder ever revealed a button-holed capsule in the locations above mentioned, unless in the instances where extensive lacerations may have followed great violence inflicted by powerful machinery.

Granting, then, the capsule to be so thick and strong above, behind, and before, as not to be ruptured during ordinary dislocations, we come to ask ourselves where is the capsule weakest—at what point is it most frequently ruptured? This place of least resistance corresponds with the lowermost quarter segment of the capsular arc; it intervenes between the two points of contact with the capsule of the subscapularis in front, and the teres minor behind. Unfortunately this weak spot corresponds with the sharpest or most prominent angle of the head of the humerus, so that when an ordinary violent force is impinged against the humeral shaft in such a direction as to give this prominence a downward tendency, the capsule ruptures, out slips the head, taking a direction downwards towards the axillary space—the direction which all authors agree the head most frequently takes.

Once the head is dislocated it may find itself a bearing at any point on the semi-circle between the coracoid process in front and the spine of the scapula behind, the precise location where it rests depending upon the counterbalanced action of muscles or sets of muscles, or the preponderating action of muscles over their antagonists. Thus, if the head rests on a right line below the glenoid fossa, I take it to be due to the even adjustment of the muscular force exerted by the subscapularis from before, and the teres minor from behind, at the same time that the supraspinatus butts the head firmly up against the triangular depression occupied by the tendon of origin of the long head of the triceps. If the head is pulled forwards, it is due to the preponderating action of the subscapularis, whereas if it is pulled backwards towards the dorsum scapulæ, the infraspinatus and teres minor preponderate. I now submit this proposition to your consideration:—

That this anatomical description renders the classification of shoulder dislocations beautifully simple. The head of the bone driven by ordinary violence leaves the capsule at the point of least resistance, viz: below. Having issued, it finds itself a bearing directly below the point of egress, or in front of it, or behind it, as the case may be, in obedience to the amount of force exerted by certain muscles. So that we can abandon as useless and perplexing the classification which recognizes subglenoid, and subcoracoid, and subpectoral, and infraclavicular, and infraspino s dislocations, since they are merely modifications of the one dislocation downwards. The head of the bone being out of socket, the next question which claims our attention is the best method for its reduction.

Some time ago when making a dissection of the shoulder-joint, I experimented as follows:—The capsule was button-holed below, and the head forced out and made to find itself a bearing downwards, forwards, or backwards as occasion required, but no matter where located it could be wound into its socket by the following manipulations. The head being forward, the forearm was flexed on the arm, and the arm on the shoulder to as near a right line as possible. By this movement the head was freed at its bearing, and the coraco-humeral ligament and the tendons of the muscles already described relaxed. Then the arm was carried upwards and forwards across the chest, its axis being slightly rotated in a direction outwards. This movement threw the head backwards towards the glenoid major and downwards towards the button-hole of the capsule. Now if the arm was rotated briskly inwards, the head glided in with a snap, and all deformity disappeared. The mechanism of the third movement I take to be as follows: By rotating inwards the coraco-humeral ligament, and the tendons inserted into the major tuberosity, viz: the supraspinatus, infraspinatus, and teres minor, were put upon the stretch, in virtue of which tension they pulled upon the capsule from above and behind, drawing apart the edges of the button-hole, at the same time that they served as rolling bars on which the head wound into its socket around the major tuberosity as a pivot. When the dislocation was backwards a reversal of these movements always resulted in reduction, and when downwards the same manipulation as practiced for luxation forwards succeeded.

With a high degree of eagerness I awaited the coming of a patient on whom to test the experiments made upon the cadaver. The test case appeared in the person of Rebecca Williams, æt 30, a hand employed in a fruit establishment on Water street. She had been walking over a wet floor with a pailful of tomatoes in her right hand, when, suddenly her feet slipped, and she fell, striking on her left shoulder. I saw her half an hour afterwards. I bared the left arm up to the shoulder, and hastily running my hand over the bones of the forearm and arm excluded fracture. Directing my attention to the joint, I noticed flattenings of the deltoid, and a depression under the acromion; also a prominence in front of the joint, and a change in the axis of the arm which threw the elbow backwards, and outwards from the side. Placing my hand on the prominence in front of the joint, I found it to be the humeral head, and on taking the shaft, and attempting motion, motion was gone. I called a friend to assist me, and directed him to hold the scapula immovable. I grasped the forearm with my left hand below the elbow, and the arm close up to the axilla with the right, flexed both on the shoulder, carried the arm upwards across the chest, rotated briskly inwards, and the ball was in its socket. Twice during the operation I heard a grating; first, when the arm was carried towards the chest, secondly when rotated inwards. The noise was heard by all present. The reduction did not occupy as much time as its description; the amount of force exerted could be furnished by an eight year old child, and the pain caused the patient was exceedingly slight.

This paper is submitted for publication with a view of stimulating surgeons to a closer study of anatomy—especially of joint anatomy. If an accurate knowledge of the tendons and ligaments around the joints prevailed, if the part they might be made to play in reducing dislocations were thoroughly understood, I am confident that reduction by manipulation, and not by great force, would be the order of the day.

ROCHESTER, March 17th, 1879.

